# Electrosensory capture during multisensory discrimination of nearby objects in the weakly electric fish *Gnathonemus petersii*

**DOI:** 10.1038/srep43665

**Published:** 2017-03-03

**Authors:** Sarah Schumacher, Theresa Burt de Perera, Gerhard von der Emde

**Affiliations:** 1 Institut für Zoologie, Universität Bonn, Endenicher Allee 11-13, 53115, Bonn, Germany; 2 Department of Zoology, University of Oxford, South Parks Road, OX1 3PS, Oxford, United Kingdom

## Abstract

Animal multisensory systems are able to cope with discrepancies in information provided by individual senses by integrating information using a weighted average of the sensory inputs. Such sensory weighting often leads to a dominance of a certain sense during particular tasks and conditions, also called sensory capture. Here we investigated the interaction of vision and active electrolocation during object discrimination in the weakly electric fish *Gnathonemus petersii.* Fish were trained to discriminate between two objects using both senses and were subsequently tested using either only vision or only the active electric sense. We found that at short range the electric sense dominates over vision, leading to a decreased ability to discriminate between objects visually when vision and electrolocation provide conflicting information. In line with visual capture in humans, we call this dominance of the electric sense *electrosensory capture*. Further, our results suggest that the fish are able to exploit the advantages of multiple senses using vision and electrolocation redundantly, synergistically and complementarily. Together our results show that by providing similar information about the environment on different spatial scales, vision and the electric sense of *G. petersii* are well attuned to each other producing a robust and flexible percept.

The vast majority of studies in the field of sensory biology have been restricted to a single modality. However, objects and environments contain inherently multimodal information, therefore animals are likely to use information from multiple sensory channels to analyse features of their environment and to guide behaviour^1^. A fundamental question in sensory biology is how multiple sensory systems operate together to produce an appropriate behavioural response. Here we aimed to investigate this question by using an object discrimination paradigm based on operant conditioning, to explore the interaction of the active electric sense and vision in the weakly electric fish *Gnathonemus petersii*.

In a multisensory system each sense provides an individual stream of information about the environment based on different physical stimuli. In order to form a robust and reliable overall percept these individual streams can be combined and integrated in different ways. The combination of different information sources can be used to increase the information input, and the integration of information from different senses about the same event or object can increase the reliability of the resulting percept^2^. However, there is inherent noise within all sensory systems; so, even if different senses provide information arising from the same source, the informational content of the inputs varies slightly. For example, the visually perceived size of an object might differ slightly from the haptically perceived size of the same object. Instead of increasing the reliability of the percept through integration, these discrepancies between the inputs of different senses might lead to a decrease of reliability if combined equally. To prevent this decrease and to obtain a coherent and reliable percept, not all senses contribute to the overall percept to the same degree. Instead the information from the different senses is integrated using a weighted average of the inputs. Studies in humans, monkeys and rats have found that this weighting depends on the reliability of the sensory inputs^3^,^4^,^5^,^6^ and is probably also based on prior experience^2^,^7^. As a result, the percept, and thus the observable behavioural output, is often dominated by a certain sense under certain conditions and during a certain task. Examples for such dominance of a sense can be seen in visual capture in humans during spatial tasks and in auditory capture during timing tasks. In humans, spatial decisions such as size estimation or source localisation are dominated by visual information, which can be observed when the sensory inputs of vision and the haptic or acoustic sense provide conflicting information^8^,^9^. On the other hand, this dominance is reversed during tasks that require temporal decisions. For example during the identification of the number of presented light flashes and beeps, the number of presented beeps will affect the number of perceived flashes^10^,^11^. Although in these and other examples one sensory input dominates, the observed behaviour is still also influenced slightly by the input of the other senses. Therefore, drastic discrepancies between the different inputs might still lead to a decrease of the reliability of the overall percept. In order to prevent this loss of reliability, integration breaks down if the discrepancies between multisensory inputs are too large and the different inputs are processed separately (segregation)^7^. The system is thus able to prevent the integration of information arising from two different events or objects.

The principles of integration through weighting of sensory inputs and segregation enable a multisensory system to obtain a robust percept and to exploit the advantages of possessing multiple information sources. For example, integration of multiple sensory inputs often leads to a *synergetic* effect, improving the behavioural response during multisensory trials compared to single sensory trials^12^,^13^,^14^,^15^. Segregated information can be *complementary*, if the information streams are tuned to particular tasks or to components of a task^16^. Furthermore, information from multiple senses could be used *redundantly*, where one sense can independently guide a behaviour but can be replaced by another if it becomes unavailable, for example at night when low light levels mean that vision cannot be used^17^.

Here, we aim to investigate these principles of multisensing by using the African weakly electric fish *Gnathonemus petersii* as a model. *G. petersii* is primarily known for its ability to orientate and communicate using its active electric sense. These fish are able to detect and discriminate objects by producing weak electric pulses in an electric organ located in the caudal peduncle (electric organ discharges (EODs))^18^,^19^. Each EOD generates an electric field around the animal. Objects within this electric field distort the spreading of the field lines, creating an electric image^20^,^21^, and these distortions can be perceived by the fish using special cutaneous electroreceptor organs^22^. Electric images provide fine scale spatial information about the shape, size and location of nearby objects and they additionally provide information about the electrical properties of objects, such as their resistance and capacitance, which inform the fish whether these objects are animated or inanimate^19^.

Besides the prominent active electric sense, *G. petersii* possess a highly specialised visual system. As an adaptation to their crepuscular or nocturnal life style and their habitat in black water streams, these fish possess a so-called grouped retina, in which the photoreceptors are packed into bundles surrounded by a tapetum lucidum^23^,^24^. This organisation of the photoreceptors improves vision under dim light and within turbid water but comes with the cost of a relatively low spatial resolution (minimal visual angle of about 3°)^25^. Since this visual system has a high temporal resolution^26^, it mainly functions to detect fast movement of bigger objects such as predators but it also enables the fish to discriminate between objects^27^.

In an earlier study^27^, we showed that *G. petersii,* which were trained to discriminate between two objects using either only the active electric sense or only vision, were capable of spontaneous cross-modal object recognition. Furthermore, when trained only with the active electric sense, electrolocation dominated over vision during object discrimination at short range. With increasing object distance this dominance of the active electric sense diminished, suggesting that the sensory inputs were weighted dynamically according to their reliability^27^. During these experiments the fish could use only single senses to learn the task, which might have influenced the hierarchy of the senses through learning. The question remains as to how vision and the active electric sense operate together during an object discrimination task under more natural conditions when both senses can be used to acquire information about the object. Is there still a hierarchy of the senses or are vision and the electric sense weighted equally, when both senses could be used during training?

Here we extended this work by investigating sensory dominance, also known as sensory capture, with fish that were trained with both senses available, by subsequently comparing their performance during visual and electrical uni-modal tests. Furthermore, by investigating the performances of fish trained (1) with both senses, (2) only with vision (intact and electrically silenced), and (3) only with the active electric sense, we were able to study additional aspects of sensory weighting, such as remapping and the influence of sensory conflict, and whether there are any advantages of using both senses to solve similar tasks, including, redundancy, synergy and complementation. Together, our experiments indicate how the active electric sense and vision operate together to produce an appropriate behavioural response during object discrimination.

## Methods

### Subjects and set up

**Figure 1:**
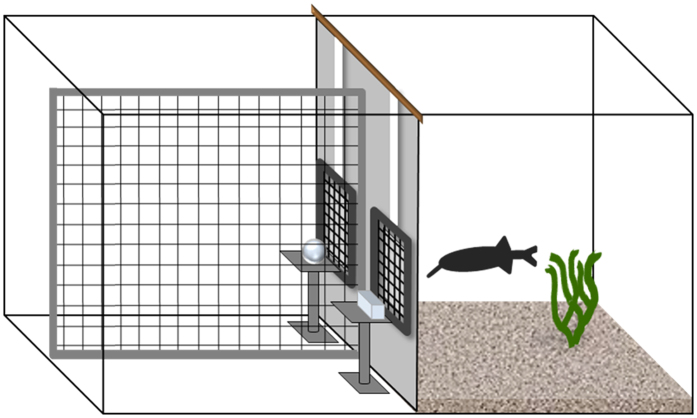
Schematic side view of the experimental tank.

The subjects were eighteen naive *G. petersii* with a standard length of 9–14 cm. The fish were kept individually in 75 cm × 40 cm × 40 cm tanks, which also served as experimental tanks. Each tank was divided into two compartments (40 cm × 40 cm and 35 cm × 40 cm) by a partition containing two gates. The bigger compartment was used as the experimental area. It was again divided into two compartments, each of which was connected with the smaller section (living area) through one gate (Fig. 1).

During the experiments objects were placed 1 cm behind the gates in the experimental area. Grids were placed behind the gates to make sure that the fish always kept the same minimum distance while inspecting the objects. These grids were made of a plastic frame stringed with cotton thread (diagonal mesh size 15 mm) and the fish had to push them aside in order to pass the gate. Most experiments were conducted under dim light conditions of 3–6 lx measured just above water level with a light probemeter (Extech instruments, Nashua, USA), which is within the range that allows optimal visual object recognition in *G. petersii*^28^. The experiments that required exclusion of visual input were conducted in complete darkness (<0.01 lx).

### Experimental procedure

The fish were trained in a two alternative forced choice procedure to discriminate between two objects that only differed in shape. At the start of each trial, the fish remained in the living area with closed gates, and the positive object (S+) and the negative object (S−) were placed behind the gates on the left or the right side of the experimental area according to a pseudorandom sequence^29^. To start the trial, the gates were opened simultaneously, and the fish was able to inspect both objects through the gates. If the fish chose to swim through the gate with the S+ behind, it received a food reward (chironomid larva), while the selection of the S− was punished by chasing the fish back into the living area. After a correct decision, the fish was given approximately 1 min to return to the living area. If the fish did not swim back within this timeframe, it was carefully forced back into the living area (without inflicting stress). When the fish was back in the living area, the gates were closed and a new trial was prepared. Each fish conducted 15–44 trials per day.

Training was considered successful when the fish reached a pre-assigned learning criterion of >75% correct choices on three consecutive training days. After the fish reached this criterion, tests were interspersed every third trial. This training to test ratio was increased to 2:2 after 3–5 days. During test trials fish were neither rewarded nor punished to prevent training effects during the tests.

### Training groups and tests

Fifteen fish were divided into three training groups of five fish each, which could use either both vision and the active electric senses (B group; fish 1–5), only vision (V group; fish 6–10) or only the active electric sense (E group; fish 11–15) to discriminate between the objects. Additionally three fish were “electrically silenced” before being trained with only vision available (S group; fish 16–18).

**Figure 2:**
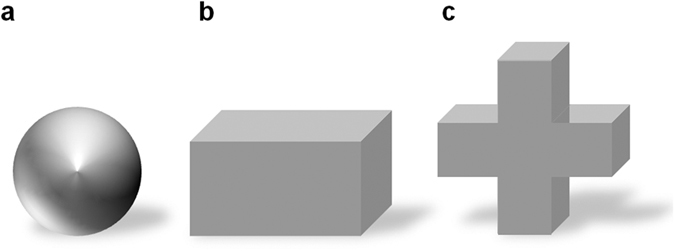
Shapes of objects used during training and tests. The objects had the same volume. The material of the objects differed for the different training groups. For the experiments with the B-group, which could use both vision and the electric sense for the discrimination task aluminium objects were presented in ambient light. During vision only training and tests, the objects were made of red coloured electrically transparent agarose. For the training with only the electric sense (E group), the aluminium objects were covered with black cotton hoods. During the uni-modal test with the electric sense, aluminium objects were encased in cubes of electrically transparent agarose and presented in complete darkness. The sphere (**a**) was used as the positive object during all experiments except for the tests with an exchanged positive object. The cuboid (**b**) and the cross (**c**) were used as negative objects during training and during the uni-modal tests.

During all experiments a sphere (Ø 3 cm) was used as the S+. In the B, V and E training group three fish each were trained with a cross (width: 4 cm, height: 4 cm, depth: 1.7 cm) and two with a cuboid (4 cm × 2.2 cm × 1.7 cm) as S− (Fig. 2). In the S training group, the cross was used as S− for two fish and the cuboid for one.

To enable the fish to use both the active electric sense and vision to learn the discrimination task, the objects that were used during training in the B group were made of aluminium and were presented under light conditions.

For training in the V group as well as in the S group, electrically transparent agarose objects were used, which had approximately the same conductivity as the surrounding tank water, therefore they were “electrically invisible” to the fish. To improve the visual perceptibility, the objects were coloured with red food colour. Red was chosen because the absorption maximum of the cones of *G. petersii* is at a wave length of 615 nm^23^. To produce the objects, red food colour (Lebensmittelpaste Rot, Deko Back, Waibstadt, Germany) was added to deionised water (conductivity: 8 μS/cm) until a conductivity of ca. 40 μS/cm was reached. Agarose powder was added in a ratio of 2 g per 100 ml liquid (increasing the conductivity to ca, 100 μS/cm) and the mixture was boiled and cast in moulds. The objects were ready to use when the agarose became stiff.

To measure whether the electrical properties of the agarose objects matched those of the tank water, the resistances of 250 ml stiff agarose and 250 ml tank water were compared. For these measurements, the measuring electrodes of a multimeter (M-3650B, Voltcraft) were injected with 5 cm distance to each other in the agarose or water. There was no measurable difference between the resistance of the agarose and that of the tank water. Additional control test were conducted to ensure that the fish were unable to use electrical input for the discrimination task (see control tests).

In the E training group, aluminium objects were used that were covered with electrically transparent hoods made of opaque black cotton fabric ensuring that the positive and the negative objects had the same outer shape. This prevented discrimination with vision and also excluded a possible influence of the lateral line system on the performance.

In the V training group, it was not possible to exclude an influence of the lateral line system without also interfering with the visual discrimination. Hence, control tests were conducted to ensure that the lateral line system was not involved in the discrimination in the B, V and S training group (see Supplementary Information Control tests).

The three fish of the S training group underwent a surgical procedure before training, during which the spinal cord was sectioned anterior to the electric organ located in the caudal peduncle. The fish were narcotised in a 100 mg/l solution of MS 222 (Acros Organics, Geel, Belgium). Once the fish were unconscious, the operation side was locally anaesthetized with Xylocain Gel (AstraZeneca GmbH, Wedel, Germany) and a dissecting needle was inserted into the vertebral canal. With slight movements of the dissecting needle the spinal cord was sectioned, so that the electric organ no longer received command signals from the brain, hence the fish were unable to produce electric signals.

#### Speed of task acquisition

To test whether the available sense influenced the speed of learning, the mean number of training days needed to reach the pre-assigned learning criterion of 75% correct choices on three consecutive training days was compared for the four different training groups. A Kolmogorov-Smirnov-test was conducted to test whether the training durations of each group was normally distributed. Afterwards a One-Way-ANOVA and a post-hoc-test with a Bonferoni-correction were conducted to compare the results of the different groups.

#### Accuracy of response

To test whether there were any effects of the available sense on the accuracy with which the fish solved the task, the performances of the different groups under their training conditions were compared at asymptote-level. The percentage of correct choices during all training trials after reaching the learning criterion was calculated for each fish. To allow statistical analyses, the percentage data were transformed using the Arcsine transformation. The results of each group were tested for normal distribution with the Kolmogorov-Smirnov-test and the groups were compared using a One-Way-ANOVA and a post-hoc-test with a Bonferoni-correction. The mean and the standard error of the mean were calculated for each group, transformed back and plotted in bar charts.

#### Uni-modal tests

After training, the fish trained with both vision and the active electric sense (B training group) were tested in uni-modal tests, during which the fish could use either only vision or only the electric sense to discriminate between the objects. During the tests, the fish were neither rewarded nor punished. The vision only tests were conducted with the red coloured electrically transparent agarose objects presented in light. To test the discrimination performance when only the active electric sense provided information about the objects and to prevent influence of other sensory systems, aluminium objects were encased in cubes of electrically transparent agarose and presented in complete darkness.

After all tests (increased conflict, feature detection, memory test and control tests) were completed, two of the fish trained with both senses (fish 2 and 5) were electrically silenced. Since these fish were used during the memory tests beforehand (see below), fish 2 had to be retrained again before it was silenced. Fish 5 never reached a performance below 70%, therefore retraining was unnecessary. Before the actual operation, a sham operation was conducted, following the same procedure as the real operation described above for the S training group, but instead of inserting the dissecting needle into the vertebral canal, only the skin was penetrated, so that the ability to produce electrical signals was not impaired. The fish were then tested again with the metal objects and with the electrically transparent red coloured agarose objects in light, to ensure that the surgical procedure had no effect on the discrimination performance. Because this was the case in all fish, the fish were then electrically silenced and tested again with only vision (red coloured electrically transparent agarose objects in light) available for the discrimination task.

With each test condition at least 30 trials were conducted. The performance of each fish during the different tests was plotted in a bar chart and tested for significant differences to the 50%-chance level using a Chi^2^-test. Exact Fisher-Tests were used to compare the performance under the different conditions for each fish.

#### Conflict between senses

During the uni-modal visual tests, vision and the active electric sense provide conflicting information about the objects (vision giving the information that objects are present and the electric sense giving the information that there are no objects). To test how the conflict between vision and the electric sense affects the discrimination performance, the intact fish of the B group, the fish of the V group and two fish of the B group (fish 2 and 5) after being electrically silenced were tested visually with visually transparent plastic screens (5 cm × 8 cm, 0.1 cm thick) placed in front of the red coloured electrically transparent agarose objects. During these tests the visual sense still provided information about the shape of the objects, while the electric sense provided information about the shape of the plastic screens, thus increasing the conflict between both senses compared to the visual tests without plastic screens. The screens were placed directly in front of the objects, so that the distance of the fish to the object and the screen was almost identical. With each fish at least 30 test trials were conducted and a Chi^2^-test was conducted to test whether the performance was significantly different from chance level. To test whether there were significant differences between the performances of the two fish trained with both senses before and after they were electrically silenced the exact Fisher-test was used.

#### Robustness of performance

To test for how long the fish were able to perform the learned discrimination task, three fish of the B group (fish 1, 2 and 5) and two fish of the V group (fish 6 and 7) were tested after four weeks without any training and subsequently once a week under the same conditions that they were trained with (except fish 5, which was tested after 6 weeks and then once every 4 weeks). On each test day, 20 trials were conducted, during which the fish received a food reward every third trial no matter which decision was made in order to maintain motivation. No punishment was used during these tests. When the fish reached a performance of less than 70% correct choices on three consecutive weeks, it was assumed that the fish were no longer able to perform the task and the tests were stopped.

### Ethical statement

The experiments were carried out in accordance with the guidelines of German Law, with the animal welfare regulations of the University of Bonn and with the “Guidelines for the treatment of animals in behavioural research and teaching”, Association for the Study of Animal Behaviour (ASAB), 2006. All procedures and methods were approved by the LANUV NRW (Landesamt für Natur, Umwelt und Verbraucherschutz Nordrhein-Westfalen, reference number: 84-02.04.2015.A444).

## Results

During training, 15 intact and 3 electrically silenced (S group) naive *G. petersii* were trained to discriminate between two differently shaped objects under different sensory conditions. Either they could use both vision and the active electric sense (B group; metal objects), only vision (V and S group; electrically transparent agarose objects) or only the active electric sense (E group; metal objects covered with opaque cotton hoods) to discriminate between the objects.

### Speed of task acquisition

**Figure 3:**
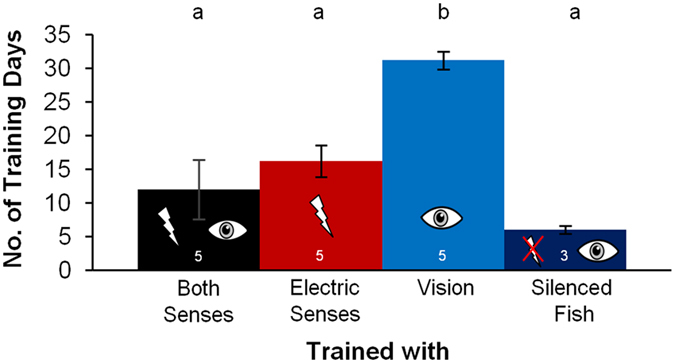
Mean number of training days the different training groups needed to reach the pre-assigned learning criterion. The error bars indicate the standard errors of mean. To test for normal distribution, the Kolmogorov-Smirnov-test was used (P > 0.05 for all groups). A One-Way-ANOVA (P < 0.001, F = 13.123) and a post-hoc-test with Bonferroni-correction were conducted to compare the different groups. The letters above the bars indicate the results of the post-hoc-test. Bars which do not differ significantly are indicated by the same letter above the bars (P > 0.05). A different letter above the bar indicates a significant difference in performance (P ≤ 0.05). The number of fish in each group is shown within the bars.

All fish, no matter which senses they could use during training, learned the discrimination task and reached the pre-assigned learning criteria of 75% correct choices on three consecutive training days. The speed of learning however differed significantly depending on the senses available (Fig. 3). The five fish of the V training group learned the discrimination task on average in 31.5 (29–36) training days, which was significantly slower than the five fish of the B training group with an average of 12.5 (4–28) training days and the five fish of the E training group with an average of 16.2 (10–23) training days. When trained under the same conditions, the three electrically silenced fish, which were not able to produce any electric signals, reached the learning criterion significantly faster than the intact V group. With an average training duration of only 6 (5–7) days these fish also learned the task slightly faster than the fish of the B and the E training groups.

### Accuracy of response

**Figure 4:**
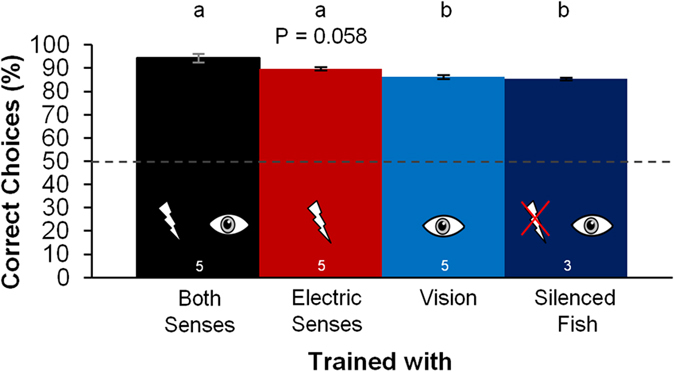
Mean accuracy of the different training groups during the training trials of the object discrimination experiments. The percentage of correct choices was calculated for each fish including all training trials after the fish reached the learning criterion. For the statistical analyses the data was arcsine transformed. The Kolmogorov-Smirnov-test was used to test for normal distribution (P > 0.05 for all groups). To compare the results of the different groups a One-Way-ANOVA (P = 0.001, F = 8.908) and post-hoc-tests with Bonferroni-correction were conducted. The mean and standard error of mean (indicated by the error bars) were calculated and back transformed. The dashed line indicates the 50% chance-level. For further description see Fig. 3.

After reaching the learning criterion, the accuracy of all of the fish increased up to at least 84% correct choices (Fig. 4). The highest accuracy at asymptote level was reached by the fish of the B training group with an average of 94.5% (90.4–99.2%), which was significantly higher than the accuracy of the V training group and the S training group. With an average accuracy of 89.8% (87.1–92.1%) the performance of the E training group was not significantly different from the results of the B group (P = 0.058) but a similar trend to a reduced performance could be observed. The accuracy of the V training group (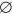 86.2% (84.6–88.2%)) and the electrically silenced fish trained under the same conditions (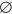 85.3% (84.1–86.0%)) were very similar.

### Uni-modal tests

After training, the fish of the B training group were tested in uni-modal tests, during which they could use only the active electric sense or only vision.

**Figure 5:**
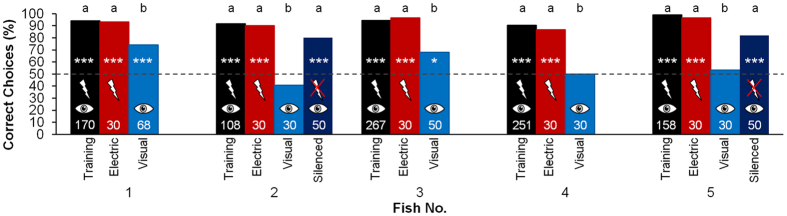
Discrimination performance of the fish trained with both senses (fish 1–5) during training (black), during uni-modal test with the active electric sense only (red) and with vision only (blue) and during visual test after being electrically silenced (fish 2 and 5; dark blue). The number of trials conducted with each condition is given within the bars. The 50%-chance level is indicated by the dashed line. To test whether the performances were significantly different from chance level, Chi^2^-tests were conducted (*P ≤ 0.05; **P ≤ 0.01; ***P ≤ 0.001). Fisher-tests were used to compare the performances under the different conditions for each fish. Bars which do not differ significantly are indicated by the same letter (**a**) above the bars (P > 0.05). A different letter (**b**) above the bar indicates a significant difference in performance (P ≤ 0.05).

All of the five fish of the B group were able to discriminate between the two objects using only the active electric sense with an accuracy of over 93% correct choices, significantly above the 50% chance level (Fig. 5). In tests, during which the fish could use only the visual sense, the performance of the individuals differed. Three (fish 2, 4 and 5) of the five fish were not able to discriminate between the objects in these tests performing just at chance level (40.7–53.3%). However two of the fish (fish 1 and 3) reached an accuracy significantly above chance level (74.2% and 68%) and were thus able to discriminate between the objects even though the accuracy was significantly lower than in training.

During the visual tests with the electrically transparent agarose objects there was a conflict between vision (providing information about the object) and the electric sense (providing the information that no object was present). The visual discrimination could have failed in the three unsuccessful fish either because the fish had not learned to use visual information for the task or because the conflict between the sensory inputs was solved in favour of the electric sense. To test between these alternatives, two of these unsuccessful fish were subsequently electrically silenced, which excluded electrical input. When subsequently tested visually under the same conditions as before, the fish reached a performance significantly above chance level (80%, 82%) and thus were now able to discriminate between the objects using only vision (Fig. 5). This means that the information for discriminating between the objects using vision must have been available but wasn’t used as long as the electric sense was working.

### Conflict between senses

To test whether the degree of conflict between vision and the active electric sense during the visual tests influences the discrimination performance, a test with a large conflict between the two senses was conducted by putting a clear plastic screen in front of the red coloured electrically transparent agarose objects. The plastic screens increased the conflict between vision and the electric sense compared to the visual tests without screens, because the fish electrically perceived the shape of the screen, while seeing the shape of the agarose objects.

**Figure 6:**
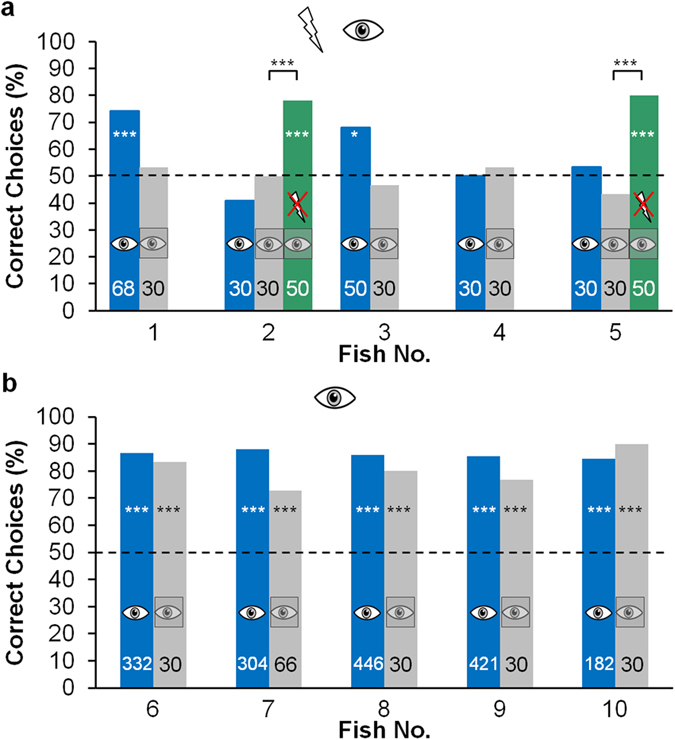
Discrimination performance of the fish trained with both sense (**a**) and the fish trained only with vision (**b**) during tests with an increased conflict between the visual and the electrical object information provided (grey). During these tests, a visually transparent plastic screen was placed in front of the red coloured electrically transparent agarose objects. Thus vision provided information about the shape of the objects, while the active electric sense provided information about the plastic screen, creating a stronger conflict between the sensory inputs compared to visual tests without plastic screens. Fish 2 and 5 were tested again after being electrically silenced (green). For reference, the performance of the fish during the visual test is shown (blue; (**a**) same data as in Fig. 3, (**b**) data from Schumacher et al 2016). An exact Fisher-Test was conducted to compare the performance during the visual tests and the test with increased conflict and the performance before and after being silenced. For further description see Fig. 5.

During these tests, none of the intact fish of the B group were able to discriminate between the objects (Fig. 6a, light blue bars). Even the two fish (fish 1 and 3), which were able to discriminate between the objects during the visual test, did not reach a performance significantly different from chance level. However after fish 2 and 5 were electrically silenced, the performance during the tests with an increased conflict significantly rose to a level significantly different from chance level (turquoise bars). These fish were now able to discriminate between the objects.

The performance of the fish of the V group during the tests with an increased conflict however did not differ from those during the visual tests and all five fish reached a performance significantly different from chance level (Fig. 6b).

### Robustness of performance

To test for how long the fish were able to perform the discrimination task without further training, three fish of the B group and two fish of the V group were tested four weeks after the last training day and thereafter once per week.

**Figure 7:**
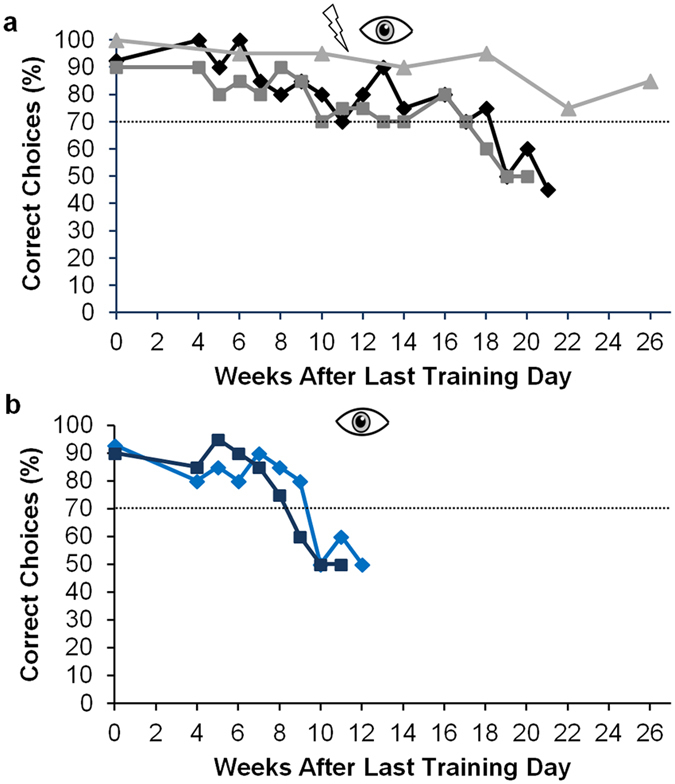
Discrimination performance of three fish (fish 1 (black), fish 2 (grey) and fish 5 (light grey)) trained with both senses (**a**) and two fish (fish 6 (blue) and fish 7 (dark blue)) trained only with vision (**b**) during tests of the robustness of performance. Fish 1, 2, 6 and 7 were tested with their trained senses after four weeks without training and thereafter once a week. Fish 5 was tested with after 6 weeks without training and from there on every four weeks. On each test day 20 trials were conducted with each fish. During test every third trial was rewarded, no matter which decision the fish made, and no punishment was applied. If the fish did not reach a performance above 70% correct choices on three consecutive test days, it was assumed that the fish had forgotten the task and tests were stopped. The dotted line indicates the threshold of 70% correct choices.

All three fish of the B group reached a performance of at least 70% correct choices up to week 17 after the last training (Fig. 7a). The performance of fish 1 and 2 dropped below 70% correct choices after 18/17 weeks. Fish 5 never reached a performance below 75% correct choices and was still able to fulfil the discrimination task after 26 weeks without training. The performance of the two fish of the V group dropped below 70% already after week 10 respectively 9 (Fig. 7b). Thus the fish trained with both senses were able to fulfil the task for nearly twice as long (or longer in case of fish 5) as the fish trained with vision alone. The comparison of the fish trained with both senses and the fish trained only with vision shows that while the performance of the fish of the B group decreased steadily with increasing time without training, the performance of the fish of the V group decreased more rapidly.

## Discussion

The presence of multiple senses allows an animal to obtain a full and flexible representation of the world. Streams of information are acquired through multiple sensory channels and are integrated at the neural level allowing the animal to respond appropriately to environmental challenges. Here, we considered the advantages of multisensing by examining the interaction of vision and the active electric sense in the weakly electric fish *G. petersii*.

### Electrosensory capture

Our results show that, at short range (1 cm distance of the objects), the active electric sense dominates over vision during an object discrimination task even if the fish were able to learn the task with both senses. In line with visual or acoustic capture in humans, we call this dominance of the active electric sense *electrosensory capture*. While all five fish trained with both senses were able to discriminate the objects electrically during the uni-modal tests at the same level of accuracy as during training with both senses, the uni-modal visual performance of those fish was significantly worse than during training. Three of the five fish were not able to discriminate between the objects at all when using only vision (Fig. 5). However, after two of those fish were electrically silenced, they were able to discriminate between the objects when using only vision. This shows that the reason that the intact fish were unable to discriminate during the visual tests (where vision alone was available for object discrimination) was not because of a general inability of the visual system to fulfil the task. Instead, this supports the hypothesis that the limited visual performance was produced by a masking effect caused by the dominance of the electric sense. During the visual test the perceptual conflict between vision (which gave the information that there was an object) and the electric sense (which gave the information that there was no object) was solved in favour of the electric sense. This led to the fish being unable, or less able to discriminate between the objects during these tests. After the fish were electrically silenced, the electric sense did not provide any information about the surrounding environment. Without any electrical input, which might overwrite the visual information, vision could be used to discriminate between the objects.

The uni-modal tests correspond with the results of fish that were trained only with the active electric sense^27^. Thus, electrosensory capture is independent of possible training effects, which might have influenced the results of the electrically trained fish. In the fish that were trained only with the active electric sense, the dominance of the electric sense might have been an effect of an overrepresentation of the electrical information during training, i.e., it might have been a training effect (remapping). However this study shows that the dominance of the electric sense remained present under more natural conditions, when the fish could use both senses to acquire and store information about the objects. This is consistent with studies on mammalian species (such as humans, monkeys and rats) that reveal that sensory inputs are weighted, which often leads to a dominance of certain senses during particular situations and tasks^3^,^4^,^5^,^6^. These results are consistent with studies in South American weakly electric fish (Gymnotidae), which also showed a dominance of the electric sense over vision during refuge-tracking and object discrimination^30^,^31^.

There are a number of potential functional explanations for the dominance of the electric sense. The active electric sense is well adapted for object discrimination. It provides detailed three-dimensional information about objects within a very short temporal scale^32^, making it a well-suited sense for object detection and discrimination. Properties like the distance or the size of an object can be extracted from only one EOD^18^,^33^. With an EOD frequency of about 30–140 Hz (during swimming and object inspection)^34^,^35^, the temporal resolution of the electric sense exceeds that of the visual sense^26^, and the fish are able to analyse their environment effectively even during fast swimming using active electrolocation. Furthermore, the electric sense is not restricted to the presence of light and is therefore available throughout day and night, which is of special importance for a nocturnal animal such as *G. petersii*. The dominance of the active electric sense might also be based upon experience. In a natural environment, it would be extremely unlikely that objects have the same conductivity as the surrounding water. Therefore, it would be unlikely that objects exist that are within the working range of the electric sense but could not be perceived electrically.

The dominance of the electric sense can also be inferred from neuroanatomical observations. A large part of the brain is occupied by areas that process the input from the electric sense (ELL, Torus semicircularis, Valvula, a.o.) while the optic tectum is reduced in comparison to other fish^36^. These immense structural differences in the brain suggest strongly that the electric sense has a central role during perception of the environment.

#### Conflict between senses

The effect of the conflict between vision and the electric sense is shown by the results of the fish that were trained with both senses, and subsequently tested with a large conflict (in comparison to the previous uni-modal visual tests) (Fig. 6a). During the visual tests without plastic screens, the conflict between vision and the electric sense lay in vision providing information about the objects while the electric sense provided the information that no object was present. These bits of information were conflicting but not irreconcilable, because there are natural condition e.g. when an object is far away, during which also only vision provides information about an object. During these tests without plastic screens, the visual information did not appear to be completely discarded and vision therefore still influenced the behaviour. When using the plastic screens, however, the electric sense and vision provided contradictory shape information. The visual sense provided the shape information of the objects and electrolocation provided shape information arising from the plastic screens (rectangular shape). The information of both senses was therefore incompatible (one object cannot have two different shapes, e.g. a cross and a rectangle, at the same time). The results show that under these conditions, all of the intact fish trained with both senses were unable to discriminate between the objects visually. Thus the increased conflict might have led to sensory segregation and a complete discarding of the visual information or at least to a further down rating of the visual information. As before, the ability of the silenced fish to fulfil the same task shows that this effect cannot be explained by a failure of the visual system.

#### Remapping

The differences in the training durations show that the dominance of the active electric sense might also influence the training performance. While the fish trained with both senses and with only the active electric sense learned the task in a similar time, the fish trained only with vision needed significantly longer to learn the task (Fig. 3). Due to the dominance of the active electric sense, visual information could have been overwritten at the start of training, which would have led to the fish being unable to discriminate between the objects when only visual object information was available. The fish could therefore not learn the task. However, due to the constant repetition of the consistent discrepancy between vision (which gave the information that there was an object) and the active electric sense (which gave the information that there was no object) during training, the system seems to be able to adjust the hierarchy of the senses (which is termed, remapping)^7^. The system could have been trained to rely on the visual information instead of relying on the usually dominant electrical input via the repeated presentation of the visual object information without any electrical object information. After the system was remapped, the visual information could have been used to learn the discrimination task. This hypothesis is supported by the results of the fish of the S group, which were trained under the same conditions as the visually trained fish after being electrically silenced. These fish learned the task significantly faster than the fish of the V group. This shows that *G. petersii* is in principal able to learn the discrimination task visually as fast as electrically and suggests that the significantly longer training duration of the visually trained intact fish might result from the additional time the system needed to be remapped.

This remapping in the visually trained fish does not seem to have been restricted to the particular conflict in information created by the red coloured electrically transparent agarose objects but seems to have led to a more general dominance of the visual sense at short range in these fish. During the tests with an increased conflict, the performance of the visually trained fish did not change compared to training (Fig. 6b). Hence the system did still rely on the visual information although the conflict was different.

The robustness of performance might also be influenced by the remapping of the system. The visually trained fish were unable to fulfil the discrimination task after 9 or 10 weeks without further training. In contrast, the fish trained with both senses were able to do so nearly twice as long or even longer (Fig. 7). This could either be explained through differences in the storage of the information or through a re-remapping of the system in the visually trained fish. Without further visual training, the everyday experience of the electric sense being more reliable at short range might have re-remapped the system so that visual discrimination at close range might have been masked again by the regained dominance of the electric sense. This hypothesis is supported by the abruptness by which the visually trained fish suddenly became unable to discriminate between the objects. Once the system was re-remapped, the fish would have been unable to fulfil the task without a constant decrease of performance. However, further experiments are necessary to test this hypothesis.

The ability to remap the weighting of the sensory inputs increases the flexibility of a multisensory system and emphasises the importance of the influence of prior experience on the weighting of sensory inputs. It allows the animal to adjust to new conditions and maintain multisensory integration at an optimal level in a variable environment.

### Multisensing: Redundancy, synergy and complementation

In addition to electrosensory capture and the flexibility within a multisensory system provided by remapping, this study shows that *G. petersii* exploits the advantages of possessing two sensory systems that can be used to solve similar tasks. We have shown that the fish are able to use the active electric sense and vision redundantly (one sense can be used as a backup for the other), synergistically (performance can be improved through multisensory integration) and complementarily (senses are tuned to particular tasks).

While the electric sense dominates during close-range object discrimination, vision can be used as a backup and is sufficient to fulfil the task alone, if the active electric sense is inoperable as was the case in the electrically silenced fish (Figs 3, 4 and 5). This redundancy has a clear adaptive value, for example if the electric organ is damaged. With its location in the caudal peduncle damages to the electric organ are possible without being lethal, and therefore the ability to use vision as a backup is highly beneficial.

The comparison of the accuracy of the discrimination performance of the four training groups suggests that there is a synergetic effect between vision and the active electric sense, since the fish trained with both senses available reached a higher discrimination accuracy than the other groups (Fig. 4). As the level of discrimination was already highly accurate when only single senses were available, this synergetic effect is rather small. However, under conditions where the discrimination performance with the single senses is near threshold level, this synergetic effect might be more important. In this way, multisensory integration would enable the fish to recognise environmental objects under suboptimal conditions and would therefore enable the fish to operate successfully under a broad spectrum of environmental settings. These results align with earlier studies that show synergetic effects during foraging and shelter seeking in *G. petersii*^37^,^38^.

The results of further tests that investigated which features the fish used to recognise the objects during the discrimination task (see Supplementary Information, feature detection tests) suggest that vision and the electric sense can also complement one another in some situations. While the fish that could use the electric sense for the discrimination task seemed to have used specific features of the objects for recognition of the negative object, the visually tested fish recognised the objects as negative as long as the general outer dimensions matched those of the known negative object (see Supplementary Fig. S3). These results correspond with the visual template matching described by Schuster and Amtsfeld^28^ and the electrical feature detection described by von der Emde and Fetz^19^. This suggests that in intact fish, the visual sense is probably not used for recognition of fine scale object information because of the low spatial resolution of the visual system^25^. Instead, acquisition of fine scale information is better provided by the active electric sense. However, due to its small working range^32^ electrical fine scale inspection of the environment is restricted to an area within the close vicinity of the fish. Thus, the visual sense might be used instead, to perceive an overview of the surroundings. This would enable the electrical information to be placed within a spatial context and would allow the fish to locate possible predators from afar. With this specific task division of vision and the active electric sense, *G. petersii* is able to use the advantages of both far and close ranging senses optimally.

## Conclusion

Together our results show that there is electrosensory capture during object discrimination at short range in *Gnathonemus petersii*. Even if the fish are able to use both senses to acquire and learn information about an object, the electric sense dominates over vision under conditions, during which both senses provide conflicting information. Nevertheless, by using both senses redundantly and complementarily and by integrating information from the senses synergistically, these fish exploit the advantages of possessing two senses, which provide similar information about the environment on different spatial scales.

## Additional Information

**How to cite this article**: Schumacher, S. *et al*. Electrosensory capture during multisensory discrimination of nearby objects in the weakly electric fish *Gnathonemus petersii.* *Sci. Rep.* **7**, 43665; doi: 10.1038/srep43665 (2017).

**Publisher's note:** Springer Nature remains neutral with regard to jurisdictional claims in published maps and institutional affiliations.

## Supplementary Material

Supplementary Information
