# Supplementary material for: Electrosensory capture during multisensory discrimination of nearby objects in the weakly electric fish Gnathonemus petersii
Source: Sci Rep. 2017 Mar 3;7:43665. doi: 10.1038/srep43665 (PMC5363222; doi:10.1038/srep43665)
Supplement: Supplementary Information [file srep43665-s1.pdf]

Supplementary Information

Electrosensory capture during multisensory discrimination of nearby objects in the weakly electric fish *Gnathonemus petersii*

Authors: Sarah Schumacher[1], Theresa Burt de Perera[2] and Gerhard von der Emde[1]

Affiliations:[1]Institut fürZoologie, Universität Bonn, EndenicherAllee 11-13, 53115 Bonn, Germany; [2]Department of Zoology, University of Oxford, South Parks Road, OX1 3PS, Oxford, United Kingdom

Correspondingauthor: Sarah Schumacher,Institut für Zoologie, Universität Bonn, Endenicher Allee 11-13, 53115 Bonn, Germany, +49 228/736159, sarah51@uni-bonn.de

Feature detection

To investigate whether vision and the electric sense are tuned to particular aspects of object discrimination, we tested which features of the objects the fish used to discriminate between them under different sensory conditions.First we tested whether both objects were used to fulfil the discrimination task or whether the discrimination task only depended on one of the objects (either the S+ or the S-). To do this, two fish of the B group (fish 1 and 2) and two fish of the V group (fish 8and 9)(all four fish were trained with the cross as negative object) were tested with: 1) the known positive object vs. a different shaped unknown neutral object (ellipsoid, Fig. S1, object G) and 2) with the known negative object vs. the ellipsoid.

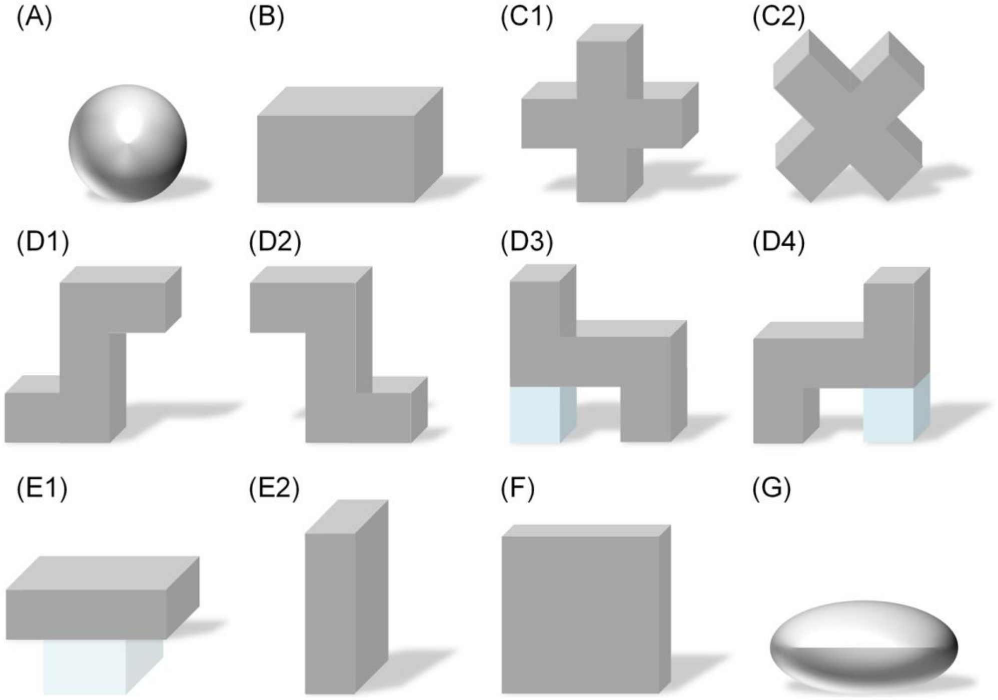

Figure S1: Shapes of objects used during the feature recognition tests**.** All objects had the same volume. The material of the objects differed for the different training groups. For the experiments with the B-group, which could use both vision and the electric sense for the discrimination task aluminium objects were presented in ambient light. During the tests with the fish trained only with vision, the objects were made of red coloured electrically transparent agarose. The sphere (A) was used as the positive object during all experiments except for the tests with an exchanged positive object. The cross (C1) was the known negative object used during. Object B, D, E, F and G were neutral and never used in experiments with these fish before. To ensure that when using objects D3, D4 or E1 the horizontal bar was presented in the same height as in the cross, small cubes of electrically and visually transparent agarose were used to lift the objects.

All fish, no matter whether trained with both senses or trained only with vision, reached a performance significantly different from chance level when the positive object was exchanged with a neutral object (see Supplementary Fig. S2 dark bars). Thus they were able to fulfil the discrimination task although the known positive object was missing. However when tested with a neutral object replacing the negative object, the performance of all tested fish dropped to chance level, showing that the fish were unable to fulfil the task without the negative object present (Fig. S2 lighter bars).This suggests that the presence of the negative object is vital for the discrimination task.Therefore the further tests to investigate which feature the fish had learned were conducted by exchanging the negative object with objects that had certain identical features as the known negative object and differed in other features. Thus if the fish was still able to discriminate between the objects, it recognised the object as negative and the object thus still contained the feature that the fish had learned during training.

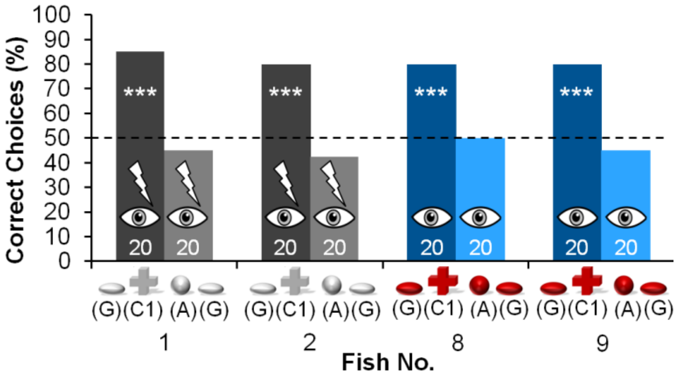

Figure S2: Discrimination performance of two fish trained with both senses (fish 1 and 2) and two fish trained with vision (fish 8 and 9) during test where either the positive object (dark bars) or the negative object (lighter bars) was exchanged by a neutral object. For further description see Fig. 3.

The feature detection tests were again conducted with fish 1 and 2 of the B group and fish 8 and 9 of the V group. During these tests,the S- was replaced by three additional unknown objects (Fig. S1 D-F), which all had the same volume as the known negative object (cross, C1). Object D consisted of the same basic elements as the cross and was presented at four different perspectives (D1-4). Depending on the perspective, the object had certain identical features as the cross, e.g. the horizontal bar or the vertical bar, with relocated features, for example, the upper and lower arm or the right and the left arm. Object E consisted only of a single bar of the cross and was presented either horizontally or vertically (E 1, 2). Object F was a cuboid with the same length and height (4 cm x 4 cm) as the cross. The known negative object (cross, C1) was also presented with a rotation of 45° (C2). Thus there were eight different test conditions that were compared with the results of the training trials with the cross. 20 trials were conducted with each condition. The percentages of correct choices with the different objects were plotted in bar charts and a Chi^2- test was used to test whether the performance was significantly different from chance level.

The results of these feature recognition tests show that all four tested fish, no matter whether trained with both senses or only with vision, were able to fulfil the task not only when the known negative object (C1) was present but also reach performances significantly different from chance level with some of the other objects, suggesting that the fish recognised certain features of the negative objects (Fig. S3). The objects the fish recognised as negative differed between the fish. Both fish of the B group were unable to fulfil the task, when only the vertical bar of the cross was present (E2) or when only the very basic dimensions of the object matched those of the cross (F), suggesting that both features together were not sufficient for the fish to recognise the objects as their negative object. While fish 2 was also unable to discriminate between the objects when only the horizontal bar was present (E1), fish 1 reached a performance significantly different from chance level with this object, suggesting that the horizontal bar was necessary for fish 1 to recognise the object. This is supported by the ability of this fish to fulfil the task when object D3 and D4 were presented instead of the cross. In both objects the horizontal bar was also unchanged. Furthermore, this fish was unable to discriminate between the objects when object D2 without an intact horizontal bar was presented. However, fish 1 also reached a performance significantly different from chance level with object D1 replacing the cross, suggesting that the presence of the right arm was crucial for the fish. Fish 2 was only able to fulfil the task when object D1 and D4 were presented. Thus, this fish might have learned a certain combination of the right arm and the upper arm.

The two fish of the V group reached a performance of at least 70% correct choices in all tests except for the tests with object E1 and 2 and thus were probably able to recognise features of the known negative object. Due to the small number of trials, a performance of 70% correct choices was not

significantly different from chance level but still it is likely that the fish were able to fulfil the task. Both fish were still able to fulfil the task when an object, which only matched the cross in its basic dimensions (F), was presented but did not reach a performance different from chance level when objects with very different dimensions (E1 and 2) replaced the negative object.

These results suggest that while there were some individual differences in the performanceof the fish, there is a trend for using more specific object features during electrical object recognition and a use of the more basic outer dimensions of the object during visual recognition.These results correspond with results from previous studies describing visual template matching and electrical feature detection.[1,2]

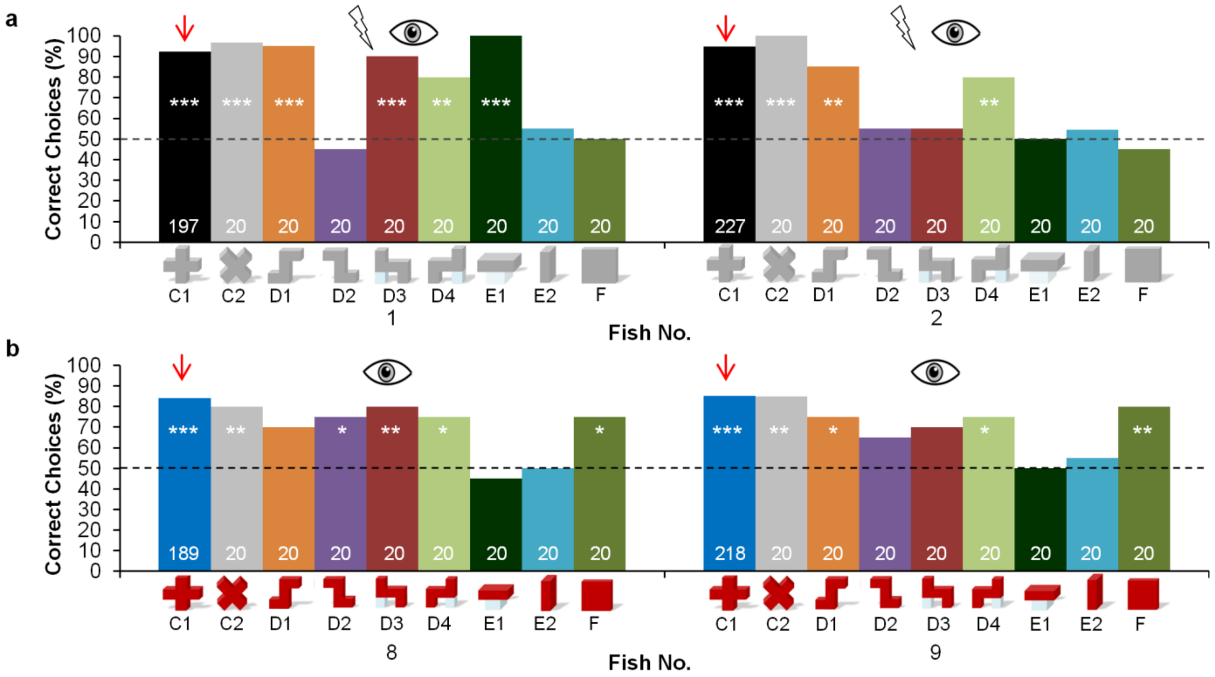

Figure S3: Discrimination performance of two fish trained with both senses (**a**) and two fish trained only with vision (**b**) during tests during which the negative object (cross, C1) was exchanged with an unknown object. The performance during training trials is shown as reference indicated by the red arrow above the bar. All objects had the same volume and were made of the same material (aluminium in **a**, red-coloured electrically transparent agarose in **b**) as the negative object. Which object was used is shown below the bars. For further description see Fig. 3.

Control tests

Electrically transparent agarose objects were used during the visual training in the V group and the S group and during the uni-modal visual tests of the B group. To ensure that the fish used no other uncontrolled sensory cues such as the lateral line system or electrical input to discriminate between these objects, control tests were conducted, during which the electrically transparent agarose objects were presented in complete darkness (< 0.01 lux). Even though the measured resistance of the agarose objects matched that of the tank water, the fish might have been able to use differences between the resistances of the objects and the water that were beyond the limits of the technical equipment to discriminate between the objects. Furthermore since the outer shape of the objects used during training and tests that involved vision was not identical, the lateral line system could possibly have influenced the discrimination performance. At least 30 control trials were conducted with the intact fish of the B group and the V group, fish 1 and 5 of the B group after being electrically silenced,

and the fish of the S group. A Chi²-test was conducted to test whether the performance during these control tests was significantly different from chance level.

None of the tested fish no matter whether intact or electrically silenced was able to fulfill the discrimination task when visionwas unavailable for the discrimination of the electrically transparent agarose objects in complete darkness (Fig. S4). The discrimination performance of all fish was close to chance level under these conditions, showing that the fish could not use other cues to discriminate between the agarose objects.

.

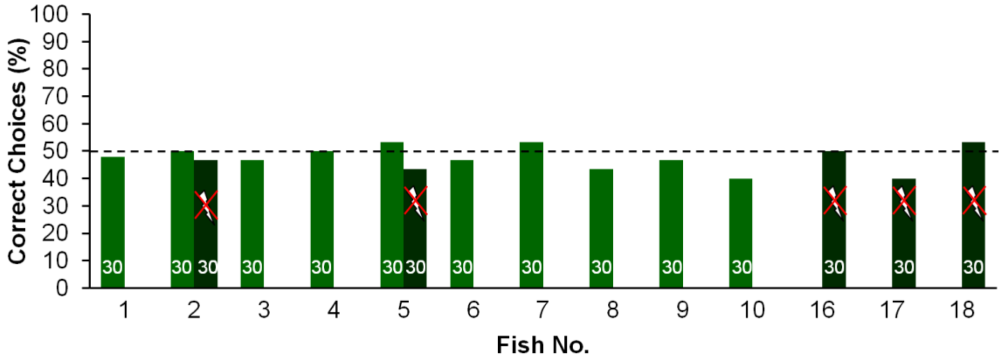

Figure S4: Discrimination performance of the intact fish trained with both senses (fish 1-5), fish 2 and 5 after being electrically silenced, the fish trained only with vision (fish 6-10, data from Schumacher et al. 2016) and the electrically silenced fish trained with vision (fish 16-18) during the dark control. To ensure that no other cues than those that were visual were used to discriminate between the red coloured electrically transparent agrose object, these trials were completed in complete darkness. For further description see Fig. 3.

A double blind control was conducted with two of the fish of the B group (fish 1 and 2) to ensure that the experimentor did not unintentionally influence the decision of the fish. During this control, an experimentor, who had previous experience with the experimental procedure but had never worked with these fish before and did not know which object was positive or negative, conducted 20 test trials with each fish. To test whether there was a significant difference between the performance with the known and the unknown experimenter the exact Fisher-test was used.

Both tested fish were able to fulfil the task when an unknown experimenter conducted the experiments, and there was no significant difference in the performance compare to the training with the known experimenter. This showed that the ability of the fish to discriminate between the objects was independent of the experimenter (Fig.S5).

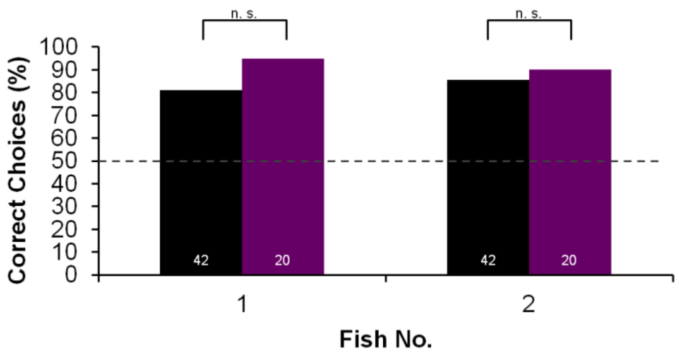

Figure S5: Discrimination performance of two fish trained with both senses (fish 1 and 2) during the double blind control with an unknown experimenter (purple). The experimenter had previous experience with the experimental procedure but did not know which object was positive and which negative. For comparison the performance with the known experimenter is given (black). An exact Fisher-test was conducted to compare the performances (n.s.: $P > 0.05$). For further description see Fig. 5.

1 Schuster, S. & Amtsfeld, S. Template-matching describes visual pattern-recognition tasks in the weakly electric fish Gnathonemus petersii. *Journal of experimental biology* **205**, 549-557 (2002).

2 von der Emde, G. & Fetz, S. Distance, shape and more: recognition of object features during active electrolocation in a weakly electric fish. *Journal of Experimental Biology* **210**, 3082-3095 (2007).
